# Supplementary material for: Impact of UGT1A1 Polymorphisms on Febrile Neutropenia in Pancreatic Cancer Patients Receiving FOLFIRINOX: A Single-Center Cohort Study
Source: Cancers (Basel). 2022 Feb 28;14(5):1244. doi: 10.3390/cancers14051244 (PMC8909027; doi:10.3390/cancers14051244)
Supplement: Supplementary file 1 [file cancers-14-01244-s001.zip › cancers-1613861-supplementary.pdf]

Supplementary Table S1. Allele frequency for UGT1A1*6 and UGT1A1*28 in 154 patients who received FOLFIRINOX for pancreatic cancer.

**Allele**	**n**	**%**
Extensive metabolizer	71	46.1
Intermediate metabolizer	66	42.9
UGT1A1 *1/*6	48	31.2
UGT1A1 *1/*28	18	11.7
Poor metabolizer	17	11.0
UGT1A1 *6/*6	5	3.2
UGT1A1 *28/*28	1	0.6
UGT1A1 *6/*28	11	7.1

Supplementary Table S2. Baseline characteristics according to UGT1A1 phenotype.

**Variables**	**Extensive metabolizer (n = 71)**	**Intermediate metabolizer (n = 66)**	**Poor metabolizer (n = 17)**	***p* value**
Age, years	61 (56-67)	62 (55-67)	63 (54.5-69)	0.845
Sex, no. (%)				
Male	47 (66.2)	38 (57.6)	10 (58.8)	0.574
Female	24 (33.8)	28 (42.4)	7 (41.2)	
ECOG PS				
0	64 (90.1)	52 (78.8)	14 (82.4)	0.203
1	7 (9.9)	14 (21.2)	3 (17.6)	
BMI, mean (kg/m2)	22.9 (21.0-25.2)	22.6 (21.4-24.8)	22.3 (20.9-26.1)	0.931
DM	20 (28.2)	25 (37.9)	5 (29.4)	
Location				
Head	34 (47.9)	40 (60.6)	9 (52.9)	0.330
Body/Tail	37 (52.1)	26 (39.4)	8 (47.1)	
Stage				
Resectable	5 (7.0)	4 (6.1)	3 (17.6)	0.627
Borderline Resectable	10 (14.1)	12 (18.2)	2 (11.8)	
Locally advanced	23 (32.4)	26 (39.4)	6 (35.3)	
Metastatic	33 (46.5)	24 (36.4)	6 (35.3)	
Laboratory characteristics				
WBC (/μL)	6860.0 (5240.0-7880.0)	7085.0 (5912.5-8472.5)	6250.0 (5050.0-8815.0)	0.575
Neutrophil (/μL)	4210.0 (3130.0-5380.0)	4140.0 (3537.5-5715.0)	4190.0 (2865.0-5310.0)	0.765
Lymphocyte (/μL)	1610.0 (1310.0-2110.0)	1665.0 (1292.5-2040.0)	1600.0 (1315.0-1925.0)	0.986
NLR	2.3 (1.8-3.4)	2.5 (1.9-3.9)	2.3 (1.6-3.2)	0.704
Hemoglobin (g/dL)	12.6 (11.6-13.7)	12.5 (11.6-13.6)	12.5 (10.3-13.2)	0.416
Platelet (10^3/μL)	229.0 (200.0-285.0)	262.0 (197.8-326.0)	269.0 (177.5-345.0)	0.646
Total bilirubin (mg/dL)	0.5 (0.4-0.8)	0.6 (0.5-1.1)	1.2 (0.8-2.4)	<0.001
AST, IU/L	19.0 (16.0-29.0)	20.5 (15.8-36.0)	27.0 (17.5-35.5)	0.410
ALT, IU/L	18.0 (12.0-33.0)	25.0 (16.0-45.5)	21.0 (16.5-45.5)	0.123
Albumin (g/dL)	4.1 (3.7-4.4)	4.1 (3.6-4.4)	4.0 (3.4-4.4)	0.537
CA 19-9 (U/mL)	125.0 (16.7-1160.0)	308.5 (41.3-1794.5)	342.0 (131.8-1618.0)	0.175

Median (interquartile range) or n (%)

BMI, body mass index; CA, carbohydrate antigen; DM, diabetes mellitus; ECOG PS, Eastern Cooperative Oncology Group performance status; FN, febrile neutropenia; NLR, neutrophil to lymphocyte ratio; UGT1A1, Uridine diphospho-glucuronosyl-transferase 1A1

Supplementary Table S3. Treatment characteristics according to UGT1A1 phenotype.

**Variables**	**Extensive metabolizer (n = 71)**	**Intermediate metabolizer (n = 66)**	**Poor metabolizer (n = 17)**	***p* value**
RDI of irinotecan (with referecne to 180 mg/m^2), median	100 (86-100)	95.5 (83.8-100)	89 (79.5-100)	0.134
RDI of oxaliplatin (with referecne to 85 mg/m2), median	100 (86-100)	97 (85.8-100)	88 (78.5-100)	0.132
RDI of 5-FU, bolus (with referecne to 400 mg/m2), median	33 (14-82)	50 (18.5-88)	63 (19-79.5)	0.509
RDI of 5-FU, infusion (with referecne to 2,400 mg/m2), median	100 (86-100)	97.5 (86-100)	91 (79.5-100)	0.400
Number of cycles administered, medain	12 (5-20)	10 (6-18)	11 (6-15)	0.726

median (interquantile range)
FN, febrile neutropenia; FU, 5-fluorouracil; RDI, relative dose intensity

Supplementary Table S4. Treatment characteristics during entire chemotherapy period.

**Variables**	**All patients (n = 154)**	**FN group (n = 31)**	**Non-FN group (n = 123)**	***p* value**
RDI of irinotecan (with referecne to 180 mg/m^2), median	97 (84.8-100)	88 (81-97)	100 (85-100)	0.007
RDI of oxaliplatin (with referecne to 85 mg/m2), median	99 (85-100)	91 (84-100)	100 (86-100)	0.023
RDI of 5-FU, bolus (with referecne to 400 mg/m2), median	44 (17-83)	30 (17-77)	46 (18-86)	0.202
RDI of 5-FU, infusion (with referecne to 2,400 mg/m2), median	98 (86-100)	92 (85-100)	100 (87-100)	0.014
Number of cycles administered, medain	11.0 (6.0-18.3)	11.0 (7.0-16.0)	11.0 (6.0-19.0)	0.872

median (interquantile range)
FN, febrile neutropenia; FU, 5-fluorouracil; RDI, relative dose intensity
